# Hemophagocytic Lymphohistiocytosis Associated with Respiratory Syncytial Virus Infection in an Immunocompetent Elderly Patient

**DOI:** 10.1155/2020/8833196

**Published:** 2020-12-08

**Authors:** Ralph Kamel, Rubal Sharma, Divya Asti, Arshpal Gill, Yevgeniy Skaradinskiy

**Affiliations:** Staten Island University Hospital, Northwell Health, 475 Seaview Avenue, Staten Island, New York 10305, NY, USA

## Abstract

Hemophagocytic lymphohistiocytosis is a serious and potentially fatal disorder characterized by excessive immune system activation. The disorder is diagnosed mainly based on laboratory, clinical, and pathologic criteria. The spectrum comprises hereditary or “primary” HLH that comprises genetically heterogeneous conditions, occurring during childhood. The secondary form presents later in life and is associated with several conditions mainly malignancy, autoimmune diseases, viral or bacterial infections, and hematological diseases. We present the case of an 80-year-old female patient who initially presented with an acute viral syndrome secondary to respiratory syncytial virus. The hospital course was complicated by disseminated intravascular coagulation and shock with multiorgan failure. Extensive workup revealed that several of the criteria for hemophagocytic lymphohistiocytosis were met. A review of literature fails to identify cases of hemophagocytic lymphohistiocytosis associated with respiratory syncytial virus in immunocompetent adults. This case report provides further insight on RSV as a possible etiologic agent associated with HLH and the importance of early recognition of this fatal disorder in RSV-positive patients who show unpredictable clinical decompensation.

## 1. Introduction

Hemophagocytic lymphohistiocytosis is a life-threatening disorder characterized by excessive activation of T lymphocytes, natural killer cells (NK cells), and macrophages resulting in a state of hypercytokinemia and subsequent organ damage [1]. The condition was first described in 1939 by Scott and Robb-Smith after analyzing a group of patients presenting with similar clinical features that included fever, lymphadenopathy, and hepatosplenomegaly that later progressed into cytopenias and eventually death [2]. HLH can be classified into 2 main groups, depending on the etiology. “Primary” or “familial” HLH is caused by mutations in genes that govern lymphocyte cytotoxicity, and “secondary” HLH is due to infections, neoplasms, and autoimmune diseases [3]. The true incidence of the disease has been difficult to calculate in adults due to diagnostic challenges, although consecutive cohort studies conducted between 1996 and 2011 and published by Parikh et al. estimated the incidence to be 1/2000 for adult critical care admissions, with the majority of patients being male, and cancer being the most common reported cause. The median age was 49 years, and the median overall survival was 2.1 months [4]. Diagnosis still represents a challenge and frequently occurs late in the disease course. The current consensus adopts the HLH-2004 criteria, and patients are diagnosed with the disease if they meet 5 out of 8 criteria that include fever, cytopenia, splenomegaly, hypertriglyceridemia, hypofibrinogenemia, hyperferritinemia, low or absent NK cell activity, elevated IL-2 receptor levels, and biopsy-proven hemophagocytosis [5]. HLH is often triggered by viral infections, and among those the most commonly reported are human immunodeficiency virus (HIV) and Epstein–Barr virus (EBV) infections [6]. Respiratory syncytial virus infection has seldom been reported in the literature as a trigger of HLH in elderly immunocompetent patients. We present the unusual case of an 80-year-old female patient diagnosed with RSV infection with subsequent dramatic clinical deterioration, who subsequently met the required number of criteria for diagnosis of HLH.

## 2. Case Presentation

We report the case of an 80-year-old female patient with a past medical history of atrial fibrillation, hypertension, hyperlipidemia, nonischemic cardiomyopathy, and chronic obstructive pulmonary disease who presented to the emergency department with 1 week of upper respiratory symptoms that progressed to chest pain, dyspnea, and nonbloody nonbilious vomiting the day prior to presentation.

On admission, she had a low-grade fever with a temperature of 100.3, and she was tachycardic and normotensive and had a normal oxygen saturation with mild tachypnea. A chest X-ray was performed and showed no consolidation, effusion, or pneumothorax.

Her complete blood count revealed leukocytosis of 19,960 (the differential count was not performed). Her basic metabolic panel was consistent with hyperkalemia, high anion gap metabolic acidosis, acute kidney injury, and elevated lactate level. Her transaminase and bilirubin levels were normal.

The rapid respiratory viral panel showed a positive respiratory syncytial virus (RSV) PCR and a negative influenza A and influenza B PCR.

The patient was started on vancomycin, azithromycin, and cefepime as well as a 5-day course of prednisone 40 mg.

The next day, her complete blood count showed worsening leukocytosis with a neutrophilic predominance along with thrombocytopenia. She was also found to have evidence of myocardial injury with elevated troponin T level. The patient was started on maintenance IV fluid. A CT scan of the abdomen and pelvis showed no evidence of abdominal or pelvic pathology.

**Figure 1:**
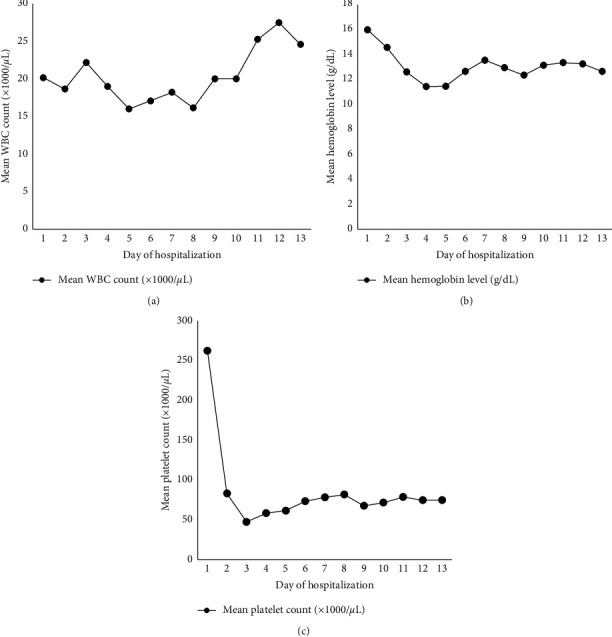
Graphs showing the variation of the mean count of each bone marrow lineage as a function of time. (a) Mean white blood cell count (×1000 cells/*μ*L) over time. Leukocytosis was noted since admission with no significant drop in white blood cell count observed during hospitalization. (b) Variation of mean hemoglobin level (g/dL) over time with acute drop in hemoglobin level observed within 3 days of admission. (c) Variation of mean platelet count (×1000 platelets/*μ*L) over time showing a significant decrease of platelet count within 3 days of admission.

Overnight, the patient became hypotensive with worsening tachycardia and increased oxygen requirements. She was started on pressor support. Her laboratory studies revealed worsening thrombocytopenia (Figure 1), hyponatremia, and acute kidney injury. She was also found to have evidence of shock liver. She was started on argatroban infusion for suspicion of heparin-induced thrombocytopenia as well as N-acetylcysteine infusion in light of a diagnosis of subfulminant liver failure.

The acute viral hepatitis panel, chronic liver disease workup, and vasculitis panel were negative. An echocardiogram revealed a decreased ejection fraction of 22%.

Hematology opinion was sought given worsening thrombocytopenia as well as the mottled cyanotic appearance of the extremities, and the differential diagnoses included disseminated intravascular coagulation (DIC), thrombotic thrombocytopenic purpura (TTP), and heparin-induced thrombocytopenia (HIT). A peripheral smear revealed the burr cells but the absence of schistocytes and blasts. Her 4T score indicated an intermediate probability of HIT with negative heparin/platelet factor IV antibody. Consequently, the argatroban infusion was stopped. The patient's coagulation panel revealed evidence of DIC, and her ferritin level was elevated at 12,760 ng/mL.

A venous and arterial dopplers of the lower and upper extremities showed no deep venous thrombosis and normal arterial flow, respectively.

Given the presence of elevated ferritin levels, the presence of pyrexia and the decreased fibrinogen level, concern about hemophagocytic lymphohistiocytosis (HLH) was raised. The patient had no splenomegaly, her triglyceride level was normal at 96 mg/dL, her IL-2 receptor level was also normal (less than 31.2 pg/mL), and her natural killer cell absolute number and percentage were within normal limits, 29 cells/*μ*L and 5% of total lymphocytes, respectively. A bone marrow biopsy was performed to rule out the presence of hemophagocytes. Her soluble CD25 level was elevated at 3990 pg/mL.

**Figure 2:**
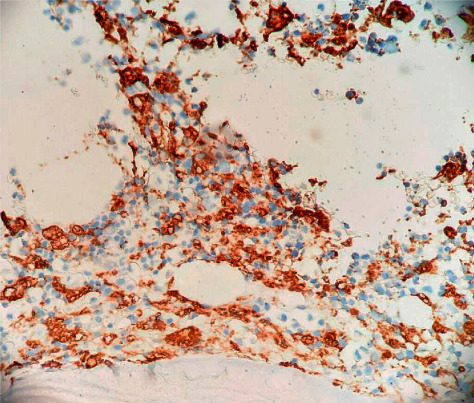
Bone marrow biopsy specimen. Immunopathology reveals positive staining for CD163, indicating moderately increased number of macrophages, some of them with hemophagocytosis.

**Figure 3:**
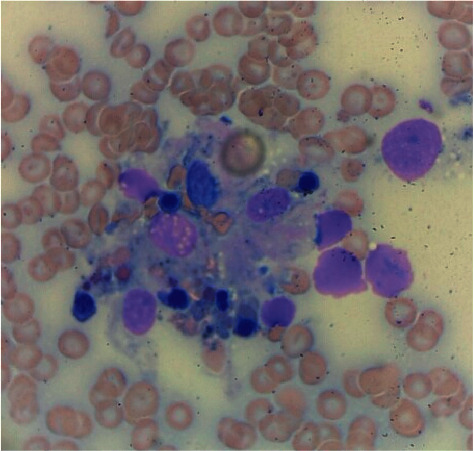
Microscopic analysis of a peripheral smear specimen with hematoxylin and eosin staining revealing the presence of macrophages with hemophagocytosis.

A flow cytometry performed on the bone marrow specimen revealed no increase in myeloid immaturity and normal myeloid antigen maturation pattern and normal lymphocyte immunophenotypic findings. Given the lack of improvement of mental status and worsening respiratory distress, a goals-of-care discussion was performed with the family, who decided to pursue comfort measures only and limit all medical interventions. The patient expired soon after. After her death, the hemopathology report of the bone marrow aspirate showed cellular bone marrow (20 to 40%) with trilineage maturing hematopoiesis with the presence of hemophagocytosis and decreased iron stores (Figures 2 and 3). 5 out of 8 criteria for the diagnosis of HLH have therefore been met.

## 3. Discussion

As presented in this case, diagnosis of HLH poses a clinical challenge and occurs late during the course of the disease.

The first definition of HLH was outlined by the Histiocyte Society in 1994 and was later revised in 2004 for the HLH-2004 trial. The revised diagnostic criteria comprise the presence of gene mutations consistent with HLH or the fulfillment of 5 of the following criteria: fever equal to or higher than 38.5°C, splenomegaly, cytopenia affecting at least 2 of the 3 lineages (hemoglobin level < 9 g/dL, platelet count < 100,000/mL, or neutrophil count < 1000/mL), hypertriglyceridemia (fasting level > 265) or hypofibrinogenemia (<150 mg/dL), presence of hemophagocytes in the reticulo-endothelial system, low or absent NK cell activity, elevated ferritin level >500 ng/mL, and elevated CD25 [7]. In this case, one of the earliest clinical manifestations was end-organ damage that presented as acute kidney injury, hypotension, myocardial injury, and fulminant hepatic failure which created additional confusion with regard to diagnosis, as septic shock and cardiogenic shock were primarily suspected early on. HLH was low on the differential diagnosis due, in part, to RSV being the infectious agent in question. As mentioned, HLH can be triggered by multiple factors and infection remains an important etiology. Meta-analyses performed previously have identified EBV, CMV, and HIV as the most common triggering viral infections [8]. Cases of HLH induced by RSV infection in adult and elderly immune-competent patients are seldom described in the literature.

Early diagnosis is critical as some therapeutic options may increase the overall survival. The first therapeutic study launched in 1994 by the Histiocyte Society used dexamethasone taper and etoposide as initial therapy followed by hematopoietic stem cell transplant or a continuation therapy combining dexamethasone, etoposide, and cyclosporine A in patients with severe, relapsed, or refractory disease awaiting bone marrow transplant. In 2004, a newer protocol that deviated from the HLH-94 protocol, called HLH-2004, added cyclosporine as part of the initial combination therapy. The newer HLH-2004 protocol was associated with nonstatistically significant reduction in pre-HSCT mortality; however, time to HSCT was significantly reduced [9]. The most recent study, an open-label, single-group study published in 2020, analyzed the efficacy of emapalumab, a human anti-interferon g, in addition with dexamethasone in children with primary HLH and showed promising results [10]. It is important to mention that most studies found in the literature have mainly focused on the pediatric population. More studies including adult patients are needed before a protocol with proven efficacy is adopted.

In our specific case, no targeted treatment was instituted for 2 main reasons: severe clinical decompensation despite supportive therapy, and final diagnosis made postmortem as only at that time had the 5 criteria been met.

## 4. Conclusion

In conclusion, our case is unique in that RSV infection in an immunocompetent elderly patient has never been described in the literature as a triggering factor for the development of HLH. This case should aware the clinicians about a possible association between RSV infection and HLH, and the importance of considering HLH as a part of the differential diagnosis in RSV patients that show dramatic clinical decompensation. It should also be mentioned that HLH is a poorly understood disease, with insidious and nonspecific presentation, and that relying on presumed associated conditions for its consideration as a differential diagnosis could potentially prove misleading.
